# 

**Published:** 1873-09

**Authors:** 


### PART III.

### EDITORIAL AND MISCELLANEOUS.

*To our Subscribers.*

☞ Look out for the red cross!

☞ We can furnish a few new subscribers with the back numbers, if they wish to have this volume complete. It will be invaluable as a book of reference.

☞ All subscribers, and others, will please write their names, post-office, county and State plainly. ☞ *Remember*, this is now required by the Postmaster-General.

☞ Should any mistake occur either in name or post-office, or should the Record fail to reach its destination, please inform the editors, who will have matters righted without delay.

☞ We invite our readers and friends every where to send us communications. Overhaul your case-books and send us your experience. Our columns are open to all.

☞ We will take the liberty to cull from private letters such extracts as may be deemed of special importance in promoting the interest of the profession.

☞ Many of the subscribers for the Record have not paid the subscription price, $2.50. Do not delay this duty, friends. ☞ It is too troublesome and expensive to forward bills; therefore, in future, we will place a red cross, as a reminder, on the journal of those who have forgotten to remit the small sum of $2.50. If we should fail to credit them in the following issue of the journal, they will please inform us. All mistakes will be corrected with pleasure. Look out for the red cross—we are looking for the money!

☞ Dr. W. T. Taylor, of Kentucky, writes: "I will be one of two hundred who will send five new subscribers, each accompanied by the cash, between this and Christmas."

☞ The above proposition we place before our readers, and pledge ourselves (if two hundred of our friends will agree to pledge themselves to send us five new subscribers each by the first of January) to enlarge the size of the journal without additional cost, and give a premium (in books, instruments or any thing else desired) to the value of seventy-five dollars to the person getting the largest number of names over five, fifty dollars to the next, and twenty-five to the next. If fifty will send us five new subscribers, the one that sends the largest number over five shall receive a premium of their own choosing worth twenty-five dollars. Go to work, friends; you will have until the first of January to get up your list of names for this volume and the next, counting from the issuing of the August number, or the first of September.

☞ In addition to the premiums already offered, each new subscriber will be entitled to Wood's Household Magazine and the superb chromo—the Yosomite Valley, 14x20—for $3.30. So, for a small sum, the RECORD, a fine LITERARY MONTHLY and a *magnificent chromo* may be had.

Any one sending us three subscribers ($7.50) will be entitled to POWELL'S FORMULARY, or WOOD'S HOUSEHOLD MAGAZINE and CHROMO, gratis.

☞ Many who have not a complete volume of the RECORD would doubtless be pleased to have a bound copy at the expiration of the year. If so, send us $4.00, and we will furnish the entire volume ready-bound for your libraries. Will our friends inform their medical friends of this?

*Errata.*

The reader will please make the following corrections in the address on Medicine and Medical Ethics, published in our August number, viz.: On page 469, for "nudentur," read "medentur"; page 470, for "Medodist," read "Methodist"; page 471, for "Rosoni," read "Rasori"; page 472, for "Father and spirits," read "Father of spirits"; page 474, for "injurious," read "ingenious."

### The Southern Medical Record.

EDITED BY

T. S. POWELL, M.D.,

AND

W. T. GOLDSMITH, M.D.

### ASSOCIATE EDITORS:

GEORGIA.

Robert Battey, M.D.
R. C. Word, M.D.
W. L. Kirkpatrick, M.D.
James A. Long, M.D.
W. L. M. Harris, M.D.
H. L Battle, M.D.
W. C. Musgrove, M D
L B. Bouchelle, M.D.
John C. Drake, M.D.
E. F. Knott, M.D.
Thomas Burdell, M.D.
E. H. W. Hunter, M.D.
V. S. Hitt, M.D.
J. E. Pope, M.D.
J. A. Hunnicutt, M.D.
A. H. Brantley, M.D.
J. J. Knott, M.D.
J. C. Wisdom, M.D.
W. W. Leake, M.D.

ALABAMA.

J. C. Knox, M.D.
Geo. F. Taylor, M.D.
J. B. Barnett, M.D.
S. M. Hogan, M.D.
D. S. Hopping, M.D.
J. T. Searcy, M.D.
S. F. Hill, M.D.

MISSISSIPPI.

C. B. Gallaway, M.D.
Edward Lea, M.D.
S. V. D. Hill, M.D.
W. B. Harvey, M.D.
J. W. M. Shattuck, M.D.
E. T. Henry, M.D.
T. P. Lockwood, M.D.
Robert Kells, M.D.

VIRGINIA.

Charles S. Lewis, M.D.
John H. Claiborne, M.D.
F. Horner, Jr., M.D.
R. J. Preston, M.D.
A. S. Payne, M.D.
J. E. Lockridge, M.D.
J. S. Wharton, M.D.
Wm. O. Owen, M.D.
Sam'l P. Ripley, M.D.
Benj. Blackford, M.D.
E. A. Drewry, M.D.
Rob B. Tunstall, M.D.
W. F. Barr, M.D.
L. B. Anderson, M.D.
S. L. Jepson, M.D., W. Va.
J. M. Lazzell, M.D.

SOUTH CAROLINA.

E. T. McSwain, M.D.
G. M. Rivers, M.D.

TENNESSEE.

J. D. Tucker, M.D.
Swan M. Burnett, M.D.

TEXAS.

S. M. Welch, M.D.
T. J. Heard, M.D.
W. A. Heard, M.D.
A. R. Kilpatrick, M.D.

ARKANSAS.

A. D. Hodge, M.D.
J. K. Hodge, M.D.

NORTH CAROLINA.

J. R. Hughes, M.D.
S. S. Satchwell, M.D.
A. B. Pierce, M.D.
H. Kelly, M.D.
Geo. A. Foote, M.D.
E. A. Anderson, M.D.
H. T. Bahnson, M.D.
Willis Alston, M.D.
Thos. F. Wood, M.D.
N. J. Pittman, M.D.

### CORRESPONDING EDITORS:

Ely Van DeWarker, M. D., New York.
John H. Weir, M.D., Illinois.
Thomas J Gallaher, M. D, Pennsylvania.
Robert Robson, M.D., Indiana.
C. C. F. Gay, M.D., New York,
*Surgeon of Buffalo General Hospital.*
W. T. Taylor, M.D , Kentucky.
B. W. Stone, M.D., Kentucky,
*Assistant Physician Western Lunatic Asylum.*
James Tyson, M.D., Philadelphia, Pa.
W. W. Leen, M.D., Philadelphia.

M. H. Henry, M.D., New York,
*Surgeon to New York Dispensary, Department of Venerial and Skin Diseases.*
Wm. R. Whitehead, M.D., Colorado Territory.
A. Patton, M.D., Indiana.
Benjamin Howard, M.D., New York.
Chas Kearns, M.D., Kentucky.
S. C. Busey, M.D., Washington, D. C.
A. W. Hobson, M.D., Arkansas.
A. W. Calhoun, M.D., now of Berlin, Prussia.
T. Curtis Smith, M.D., Ohio.
Geo. Strawbridge, M.D., Philadelphia.

☞ *All Business Letters should be addressed to POWELL & GOLDSMITH, Post Office Box* 527, *Atlanta, Georgia.*

